# Management of a post-coital recto vaginal fistula at the Douala Gyneco-Obstetric and Pediatric Hospital: a case report

**DOI:** 10.11604/pamj.2020.36.151.23281

**Published:** 2020-07-03

**Authors:** Alphonse Nyong Ngalame, Armand Talom Kamga, Rakya Inna, Darolles Wekam Mwadjie, Emile Telesphore Mboudou

**Affiliations:** 1 Douala Gyneco-Obstetric and Pediatric Hospital, Douala, Cameroon,; 2 Faculty of Medicine and Biomedical Sciences, University of Yaounde I, Yaounde, Cameroon

**Keywords:** Management, recto vaginal fistula, post-coital

## Abstract

Recto vaginal fistula can be secondary to various and multiple causes. However, intercourse is an exceptional cause. The objective of this work is to expose its clinical, therapeutic and prognostic particularities. We report the case of rectovaginal fistula in a 29-year-old patient, following consensual sex. She underwent posterior colpoperineorraphy under spinal anesthesia, with a favorable outcome. Post-coital recto vaginal fistula is a stigmatizing pathology responsible for polymorphic complications. Prompt care can improve quality of life and the obstetrical prognosis of the patient.

## Introduction

Post-coital recto vaginal fistula (RVF) which is an abnormal communication between the rectal and vaginal mucosae following sexual intercourse, is a rare but stigmatizing condition. It is usually associated with minimal or moderate per vaginal bleeding. Very few cases reported in the literature to date. In Nigeria, post-coital RVF represent 0.7 cases per 1000 gynecological emergencies [1]. In Ethiopia, a total of 91 cases of RVF were reported over a period of 6 years, with all linked to consented sexual intercourse and rape [2]. The main causes of RVF are obstetrical, followed by genital cancer, radiotherapy, Crohn's disease and anal abscess [3,4].

## Patient and observation

**Figure 1:**
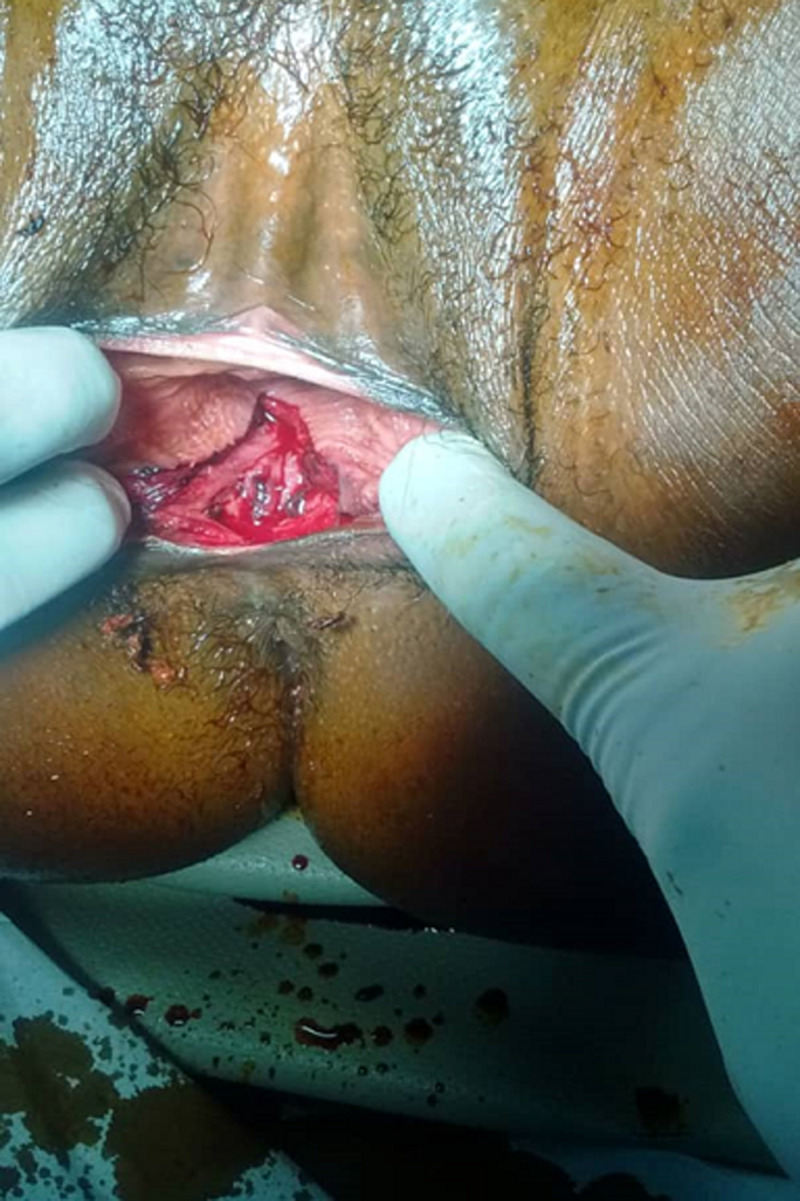
exposure of the different vulvo-perineal layers

We report the case of a post-coital recto vaginal fistula in a 29-year-old patient, single, veterinary nurse, nulliparous and desiring fertility for 3 years now. She was referred to us from a district hospital by her attending physician for better management of a rectovaginal fistula, which occurred 6 hours before her admission following vigorous vaginal sexual intercourse and consented to in the supine position, according to the patient. There followed a vaginal bleeding which had motivated a first consultation in this hospital where she had received intravenous fluids with 500ml of Ringer´s lactate and the placement of a vaginal packing. Faced with the unavailability of the gynecologist on duty, the patient was immediately referred to us. Upon arrival, she complained of perineal pain, with a blood pressure of 123/87mmHg and a pulse rate of 113 beats per minute. The conjunctivae were pink. The vulva was stained with bright red blood with packing in place. When packing was removed, we discovered a complete tear at the level of the posterior vulva (Figure 1). On speculum examination, the cervix was anterior, macroscopically normal with no lesion at the posterior fornix. On vaginal examination, the cervix was anterior, long, thick and closed, the uterus was of normal size, with no lesion at the vaginal fornixes. We uncovered a 3cm longitudinal tear communicating totally between both rectal and vaginal mucosae at the lower third of the recto-vaginal wall. Its apex was approximately 3-4cm from the posterior fornix.

**Figure 2:**
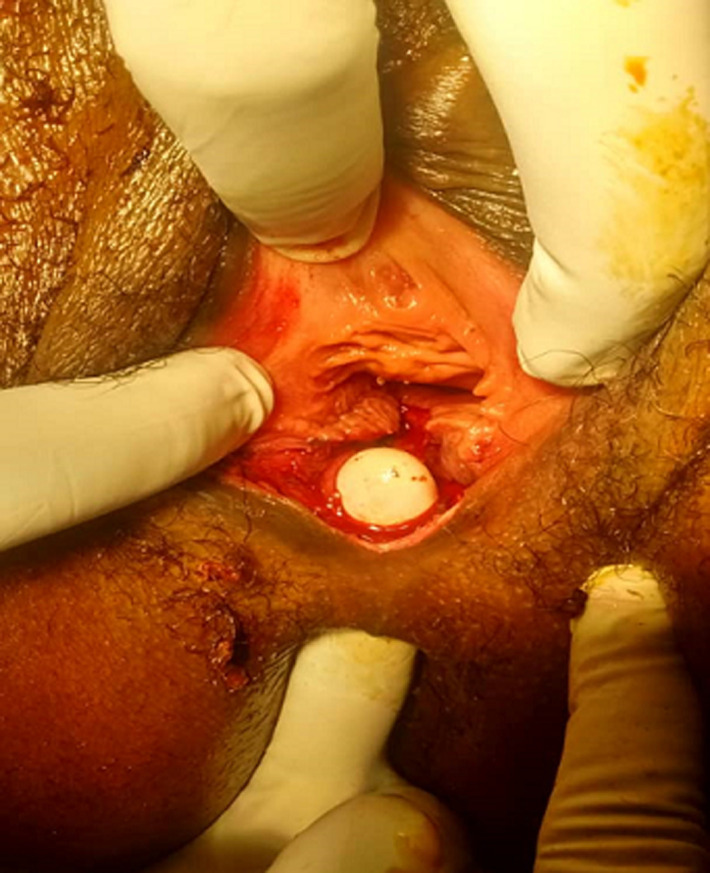
recto vaginal fistula

**Figure 3:**
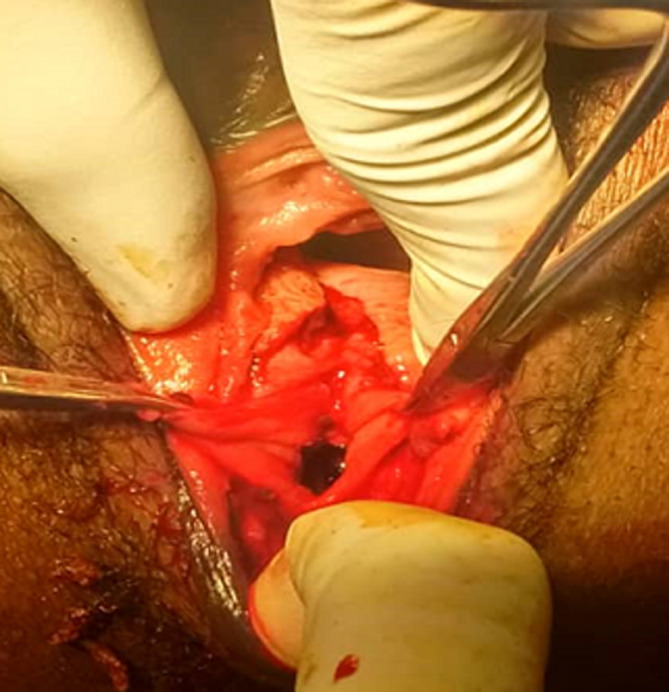
exposure of torn rectal mucosa

**Figure 4:**
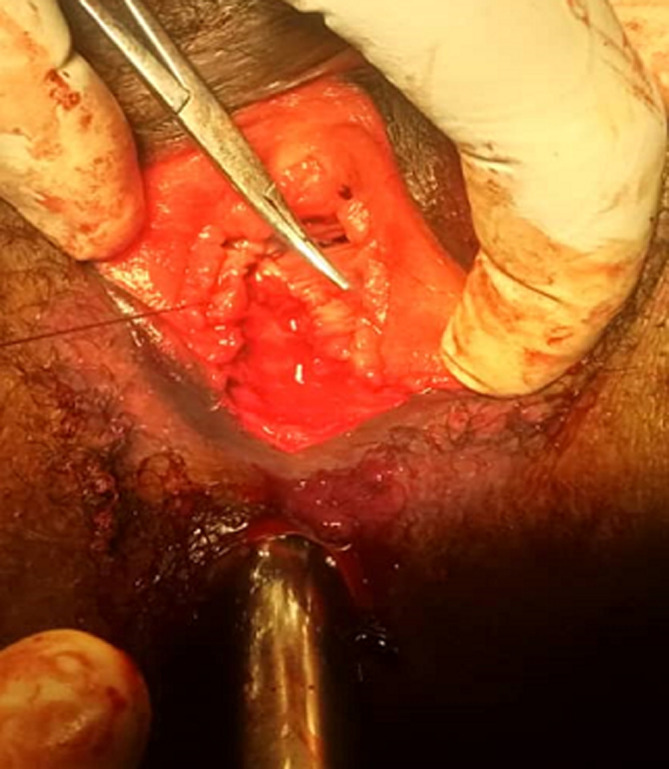
repair of rectal mucosa

**Figure 5:**
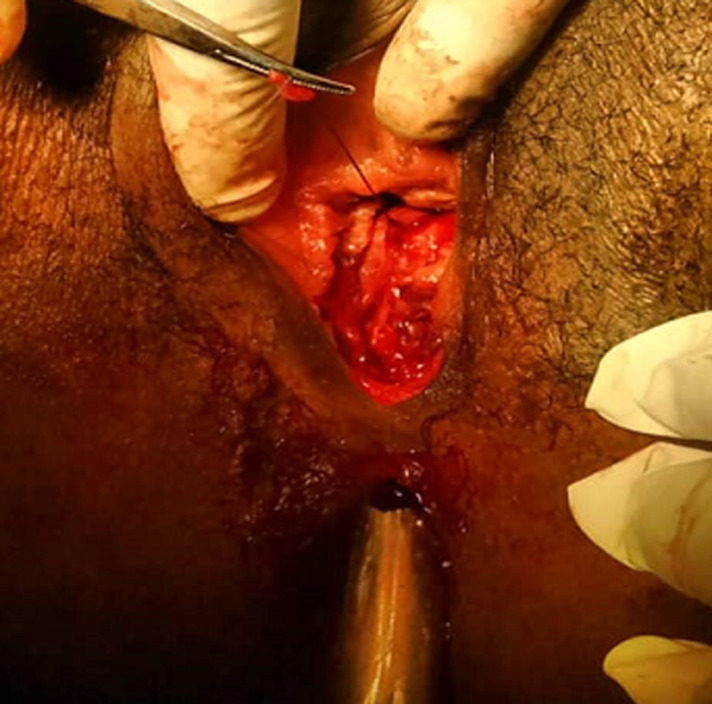
repair of the vaginal mucosa

**Figure 6:**
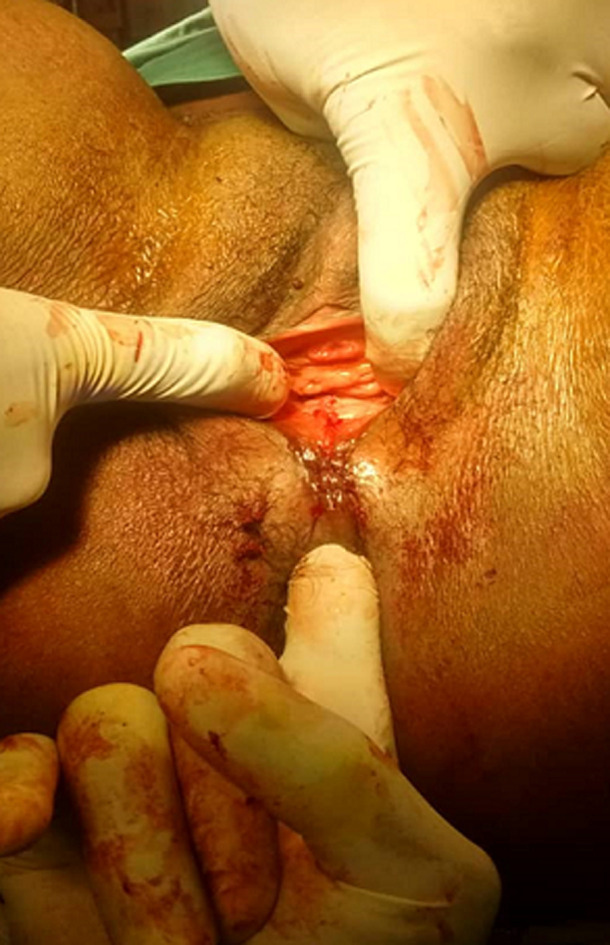
end result after posterior colpo-perineorrhaphy

**Figure 7:**
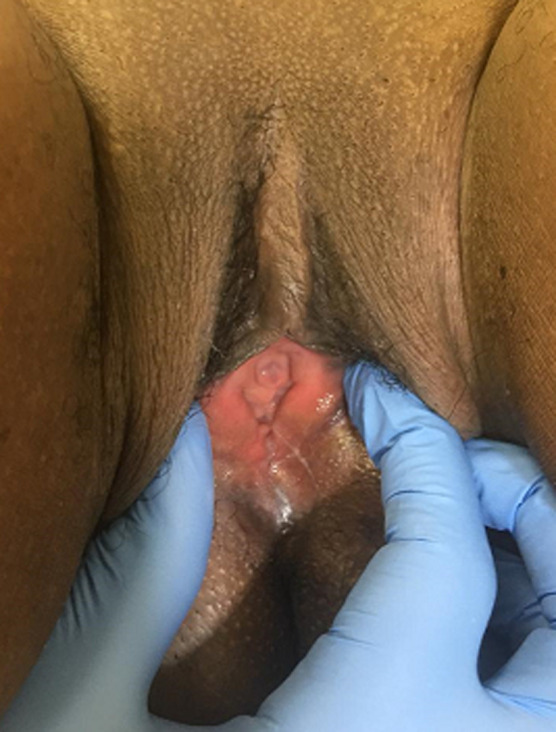
complete healing on day 60 post operatory

The external anal sphincter was intact (Figure 2). We concluded with a post-coital recto vaginal fistula of the lower third. The management consisted, after counseling the patient, in a posterior colpoperineorraphy in the operating room under spinal anesthesia. We equally offered her psycho-social support going forward. The operating procedures consisted in: betadine swab of perineum and vagina, placement of a size 12 Hegar´s dilator in the rectum, suturing of the rectal mucosa with absorbable vicryl No. 2/0 (Figure 3 and Figure 4), then a successive suture of the vaginal mucosa and of the cutaneous plane of the perineum with vicryl 0 (Figure 5). After satisfactory control of the hemostasis and checked for the solidity of the repair, we proceeded to a vaginal toileting (Figure 6). Early post-operatory follow-up was simple. She was discharge the next day under; gynecological betadine ovules and liquid for Sitz bath, amoxicillin-clavulanic acid 1g/12h and paracetamol 1g/8h orally. We also gave her diet and nutrition counseling to prevent constipation and had to suspend any sexual intercourse for the following 6 months. Her appointments for clinical review after 1 week and two months post-operatory were uneventful and satisfactory (Figure 7).

## Discussion

Post-coital RVF is a rare entity: 0.7/1000 gynecological emergencies in Nigeria [1], 91 cases in Ethiopia [2] and 32 cases reported in Senegal [5]. According to the available body of literature, the predisposing factors for post-coital RVF are; young age (15-30 years), parity (0-1), supine position during sexual intercourse, first intercourse, rough intercourse, peno-vaginal disproportion, inadequate physical and emotional preparation of the woman for intercourse and intercourse during the puerperal period [5-8]. Our patient was a 29-year-old nulliparous woman who just had consented though violent intercourse in the supine position. She has had multiple previous sexual relationships with the same partner, which permitted us to rule out the possibility of peno-vaginal disproportion. The most common site of vaginal injuries at coitus is the vaginal vault particularly the posterior fornix. Other sites include; right fornix, left fornix and lower vagina [9]. Our patient presented with a post coital recto vaginal fistula of the lower third. The diagnosis of post-coital RVF is essentially based on clinical examination, as was the case in our patient. However, there are methods of paraclinical investigations when the clinical diagnosis is not obvious. We can cite; colonoscopy, barium enema, computed tomography, magnetic resonance imaging [10]. An anorectal ultrasound may also be used to check the integrity of the anal sphincter. All these were not necessary in our patient due to the active bleeding and easy access of the lesion without external anal sphincter involvement. Surgical repair of post-coital RVF can be done either locally (by a trans-vaginal or trans-rectal approaches) or by a trans-abdominal approach [10]. The trans-abdominal approach is generally indicated for upper fistulas secondary to Crohn's disease, cancer or radiotherapy [10]. In our patient we proceeded to a transvaginal approach since she had a lower fistula. Post-operative medical care involves; local perineal care with antiseptics, oral antibiotics to safeguard against superinfection, analgesics, dietary counselling to prevent constipation, abstain from sex and psychosocial support of the couple.

## Conclusion

Post-coital recto vaginal fistula is a rare entity. However, it can cause serious damage that can alter the patient's quality of life in the medium and long term, as well as her obstetrical prognosis. In the face of any post coital vaginal trauma, a careful clinical examination would be enough to start treatment quickly and thus improve the prognosis.
